# Cheshire County Asylum at Macclesfield

**Published:** 1893-05-06

**Authors:** 


### THE STORY OF THE INSANE FROM YEAR TO YEAR.

(Fifth Series.)

### CHESHIRE COUNTY ASYLUM AT MACCLESFIELD.

This asylum came of age last year, and it now presents us with its twenty-first annual report. It increased its numbers from 539 to 570. During the year it admitted 138 patients. Forty-four were discharged recovered, and the same number died. The percentage of recoveries is 34·9, and the deaths 7 5. Hereditary tendency and drink taken together are responsible for 48 of the admissions. Domestic trouble caused the insanity in 11 cases, and epilepsy in 12. In eight cases consumption was the cause of death, and it complicated two others, while three of the deaths were from dysentery. Regarding the latter, Dr. Sheldon says, "There is no certain knowledge as to its origin; but I strongly suspect that it is due to overcrowding or imperfect ventilation, which is practically the same thing." Possibly Dr. Sheldon is right; but we believe it has broken out in well-ventilated asylums, and we feel more inclined to echo his first remark, that there is no certain knowledge of its origin. Reference to the new Lunacy Act is thus made. "Lapse of time has not brought about any modification of the unfavourable opinion almost unanimously expressed of this act." Dr. Sheldon further states that he looks upon the "control by the Government Auditor as likely to be fraught with serious consequences to the interests of the insane unless administered with something of discretion." Such are the opinions, expressed freely and unreservedly by medical superintendents; but there is much likelihood of want of result unless concerted action be taken whereby the eyes of our legislators may be opened and this Act swept off the statute book giving place to a new one. An additional assistant medical officer has been appointed, and the appointment is limited to three years. This limitation of the term of office is probably destined to become general in asylums, but we think a three years' limit somewhat short. The visitors say the condition of the asylum is satisfactory, and they have pleasure in recording their continued satisfaction with Dr. Sheldon's management." The weekly cost of maintenance is a fraction under 9s.

